# Growth parameters and IGF-1 axis biomarkers differ among omnivorous, vegetarian, and vegan children

**DOI:** 10.1007/s00394-026-04085-6

**Published:** 2026-08-29

**Authors:** Inola E. Allner, Wieland Kiess, Jürgen Kratzsch, Mandy Vogel

**Affiliations:** 1 LIFE Child, Center for Pediatric Research, University Hospital for Children and Adolescents, Faculty of Medicine, Leipzig University, Philipp-Rosenthal-Str. 27, 04103 Leipzig, Germany; 2 German Center for Child and Adolescent Health (DZKJ), Partner Site Leipzig/Dresden, Leipzig, Germany; 3 Institute of Laboratory Medicine, Clinical Chemistry, and Molecular Diagnostics (ILM), University of Leipzig, Liebigstrasse 27, 04103 Leipzig, Germany

**Keywords:** Adolescent, Body growth, Diet, vegan, Diet, vegetarian, Insulin-like growth factor binding protein 3, Insulin-like growth factor I

## Abstract

**Purpose:**

Whereas an increasing number of families choose a vegan or a vegetarian diet, the effects of a plant-based nutrition on height and BMI of children and their IGF-1- and IGFBP-3-serum concentrations as measures for growth and development are not entirely described.

**Methods:**

7344 serum samples were investigated from 2,508 healthy participants in the age of 3 months to 19 years. Anthropometric measurements as well as IGF-1-, and IGFBP-3-serum concentrations were compared between dietary groups using optimal 1:3 pair matching (from the age of 10).

**Results:**

Vegan children were shorter than vegetarians after adjustment for pubertal status (β = −0.59, *p* = 0.006) but the height of vegetarians was not different from omnivores. Vegans showed lower BMI-SDS than vegetarians, most prominently in the TS-adjusted model (β = −1.01, *p* = 0.009). Omnivores had the highest BMI-SDS (0.32 SDS). Vegans had lower IGF-1 levels than vegetarians and omnivores in the age-adjusted model (ß = −0.52, *p* = 0.031). Vegetarians exhibited lower IGF-1 concentrations than omnivores in age- and TS- adjusted models. Vegans showed significantly lower IGFBP-3-SDS than vegetarians in TS-adjusted models (β = −0.75, *p* = 0.042). IGFBP-3 values were lower in vegetarians than in omnivores after adjustment for age (β = −0.18, *p* = 0.005).

**Conclusion:**

Children following a vegan diet are significantly smaller and have a lower BMI than their peers. Both, vegetarian and vegan diets are associated with lower BMIs and lower IGF-1 and IGFBP-3 values. These findings highlight the potential relevance of dietary composition for growth childhood.

**Clinical trial registration:**

LIFE Child is registered with Clinical Trials.gov (NCT02550236) since October 2010. https://clinicaltrials.gov/study/NCT02550236.

**Supplementary Information:**

The online version contains supplementary material available at https://doi.org/10.1007/s00394-026-04085-6.

## Introduction

 The number of people following a vegetarian diet increases all around the world, especially in Western countries [1]. Research indicates that more individuals are adopting vegetarian diets, mainly due to health concerns. The second most common reasons are ethical considerations regarding animal welfare and growing awareness of the environmental impacts of meat consumption [2].

Consequently, children are more frequently being raised on these diets. This development prompts important questions regarding the suitability of vegetarian and especially vegan diets during childhood and adolescence. Adequate nutrient intake is critical during this stage of life, as the body undergoes significant growth and developmental processes. Energy, macro- and micro-nutrients must be provided in sufficient quantity and quality to support healthy physical and cognitive development [3, 4].

Hebbelinck et al. [5] reported a significantly reduced caloric intake among vegetarian children compared to age- and sex-specific reference values. The most pronounced differences were observed in some 15-year-olds, whose energy intake reached only 66% (boys) and 51% (girls) of the respective reference levels. In contrast, Sutter et al. [6] found no differences in total energy intake between vegan and omnivorous children. However, the composition of nutrient intake varied: omnivores consumed higher amounts of protein, fat, and added sugars, whereas vegans tended to consume more complex carbohydrates and dietary fiber. The German VeChi Youth Study [7] likewise concluded that omnivores consumed more protein.

Important mediator variables between diet and anthropometric outcomes include the insulin-like growth factor 1 (IGF-1) and its main binding protein termed insulin-like growth factor-binding protein 3 (IGFBP-3).

Within tissues, IGF-1 initiates growth-promoting and anti-apoptotic effects [8] and is involved in both local and systemic growth regulation [9]. It promotes bone and muscle growth [10], while also supporting adipocyte differentiation and lipid accumulation in vitro [11]. In serum, up to 90% of circulating IGF-1 is bound by IGFBP-3. This interaction decreases its degradation rate, thereby extending the half-life [12].

 IGF-1 is positively associated with body mass index (BMI), weight, height and fat-free mass [13–15]. However, there is also an association between children with obesity or adiposity and IGF-1 [14, 16]. Furthermore, multiple nutritional factors influence serum levels of IGF-1. In addition to the total energy intake, the amount of protein consumed is determining [17, 18]. Especially protein of animal origin is a crucial factor [19]. Milk protein in particular is strongly associated with higher IGF-1 levels [18]. Animal proteins in general provide a complete amino acid profile, containing all nine essential amino acids in adequate amounts. In addition, they have a higher biological value as a measure of protein quality, indicating the efficiency with which absorbed amino acids are utilized for protein synthesis [20]. Most plant proteins lack a complete amino acid profile, often being limited in one or more essential amino acids. A positive association was found between the intake of essential amino acids and IGF-1 concentrations [21]. Furthermore, consuming not enough essential amino acids can lead to stunting growth and development in children [22].

Therefore, we hypothesized that vegetarian and vegan diets in children may be associated with lower IGF-1 and IGFBP-3 concentrations. Furthermore, we assumed that participants adhering to a vegetarian or vegan diet have a lower bmi and reduced height standard deviation scores (SDS). By investigating these associations, this study aims to contribute to the current understanding of how different dietary patterns may relate to growth and endocrine markers in children. These insights may help to inform clinical awareness and guide future research on the nutritional adequacy and potential long-term implications of plant-based diets in pediatric populations.

## Methods

### Study design

All data were collected within the LIFE Child Study. LIFE Child is a prospective, population-based cohort study that has been conducted at the Research Center for Civilization Diseases in Leipzig since 2011. Subjects from the 24th week of pregnancy up to children aged 16 are included in the study and followed up to the age of 20 [23]. The study aims are to observe healthy development and to identify adverse and advantageous metabolic, genetic and environmental factors [24].

Informed written consent was provided by the parents or legal guardians. Children aged 12 years or older additionally provided written assent. However, participation in every procedure and test remained voluntary.

The LIFE Child Study follows the principles established in the Declaration of Helsinki. The study protocol was approved by the Ethical Committee of the Medical Faculty of the University of Leipzig (Registration No. 477-19-ek). The Ethics Committee is registered as an institutional review board with the Office for Human Research Protection (IORG0001320 and IRB00001750).LIFE Child is registered with Clinical Trials.gov (NCT02550236).

### Study population

We included all children and adolescents with data on serum IGF-1, serum IGFBP-3, and the necessary dietary information. Children and adolescents were excluded from the study if they had received medication interacting with the immune system or hypophysial, thyroid, or sex hormones. Furthermore, neurogenic drugs, insulin treatment, digestive, and anti-diabetic medications led to exclusion. Finally, 7344 out of 7653 available serum samples from 2508 subjects were included.

Overall, 98% of the study population were of Caucasian ethnicity. Because of the very low number of vegetarians and vegans at younger ages, we restricted our main analyses to the subjects aged 10 or older.

### Measures

#### Dietary information

At each visit, the children, and, before the age of 10.5, their parents, were asked about their habitual dietary pattern. Based on these self-reported data, their diet was categorized as omnivore, vegetarian, or vegan, which defines the main study groups for subsequent analyses.

Participants were classified as omnivorous if no dietary restrictions regarding the consumption of animal-derived products were reported. Vegetarian participants were defined as those who excluded meat and fish from their diet, while vegan participants additionally excluded all animal-derived products like eggs and diary products.

Follow-up visits were conducted with a minimum interval of one year between measurements. The current dietary status at each visit was reported at the same day as the corresponding blood withdrawal and anthropometric assessment took place. Thus, dietary classification reflects the self-reported current dietary pattern at the time of each specific measurement. This approach ensured temporal alignment between exposure (dietary pattern) and outcome measures within each study visit.

#### Anthropometry

The weight (body scale “Seca701”,seca GmbH & Co.KG, Hamburg, Germany, accurate to the nearest 50 g) and height (body measuring device “Stadiometer Dr. Keller I”, Längenmesstechnik GmbH, Limbach-Oberfrohna, Germany) of the participants were measured at each study visit by the trained assistants. The BMI was calculated and BMI and height were transformed into age- and sex-adjusted standard deviation scores (SDS) according to the S3 guidelines of the German Working Group of Childhood and Adolescent Obesity and the German Society of Pediatrics and Adolescent Medicine [25]. Pubertal stage was assessed according to Tanner [26, 27].

#### Laboratory assessment

Blood samples were collected through venipuncture, ensuring proper aseptic technique to minimize contamination and ensure the integrity of the samples for subsequent analysis. The quantitative determination of IGF-I and IGFBP-3 concentrations in human serum was carried out using a sensitive chemiluminescent immunoassay. The analyses were performed on the IDS-iSYS Multi-Discipline Automated System (Immunodiagnostic Systems GmbH, Frankfurt am Main, Germany), a fully automated platform designed for high-sensitivity chemiluminescent immunoassays. All measurements were conducted according to the manufacturer’s instructions. Interassay CVs ranged from 2.1% to 7.2% at IGF-I concentrations between 27.9 and 919 ng/mL (130–162 runs) and from 5.2% to 10.0% at IGFBP-3 concentrations between 1800 and 5498 ng/mL (135–137 runs). The IGF-1-, and IGFBP-3-SD scores were calculated using age- and sex-specific reference data from the Life Child cohort.

### Statistical analyses

Descriptive analyses are given as medians with interquartile ranges for continuous variables and absolute and relative frequencies for categorical variables. Both IGF-1 and IGFBP-3 were transformed into age- and sex-adjusted as well as Tanner Stage (TS)- and sex-adjusted standard deviation scores.

The main analysis consisted of the two main comparisons: vegetarian vs. omnivore diet and vegan vs. vegetarian diet using optimal pair matching minimizing within-pair distances across the whole sample. For both analyses, two separate 1:3 matchings were conducted: one based on sex and age, and another based on sex and TS. Generalized estimating equations (GEE) with a Gaussian error family and exchangeable correlation structure were used to estimate associations between diet groups (predictor) and IGF-1-SDS and IGFBP-3-SDS (outcomes)(both age- and TS-adjusted). As matching was performed on sex, age, and Tanner stage, these variables were not additionally included in the regression models. Matching variables are inherently controlled for by the study design and therefore do not require further adjustment in the generalized estimating equations (GEE) analyses.

BMI was not included as a covariate, as it may act as a mediator in the relationship between dietary pattern and IGF-1 levels (i.e., dietary patterns may be associated with BMI, which in turn is associated with IGF-1). Adjusting for a mediator could introduce post-treatment bias and potentially distort the estimated association. Moreover, BMI-SDS and IGF-1 levels may be influenced by shared unmeasured factors (e.g., physical activity), such that BMI-SDS could act as a collider. Adjustment for BMI-SDS under these conditions may therefore introduce additional bias. For these reasons, BMI-SDS was not included in the models.

Results were presented visually and reported as group differences (ß) and p-values. As a sensitivity analyses, we re-estimated all models using an independence correlation structure (equivalent to cluster-robust OLS) to assess the stability and robustness of the observed effects. All analyses were carried out using R (version 4.5.0) [28]. The significance level was set to α= 0.05.

## Results

### Frequencies of vegetarians and vegans

**Fig. 1:**
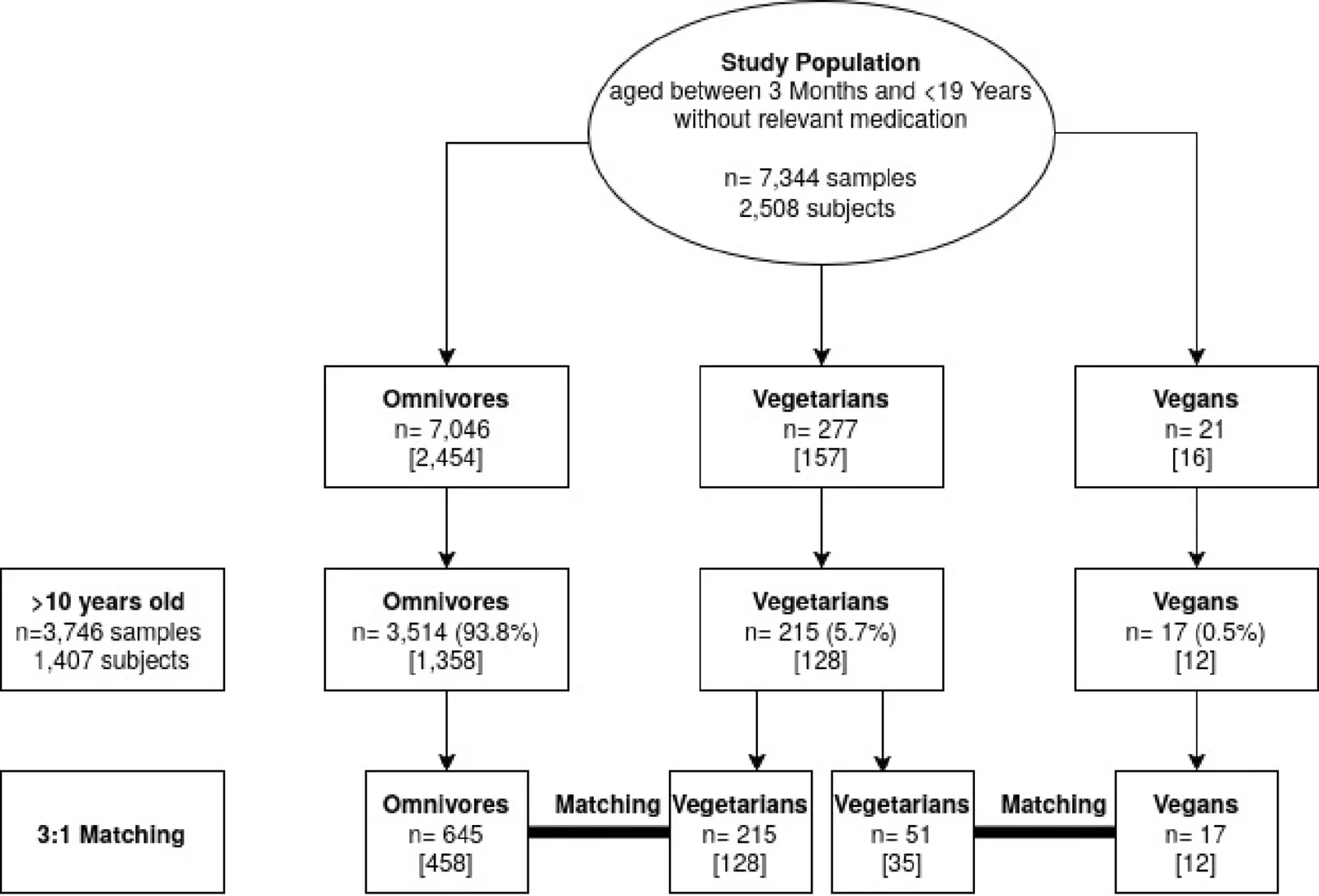
Flowchart illustrating the selection and allocation of participants across dietary groups and analytical subgroups. Numbers indicate the total number of blood samples, with the corresponding number of unique participants shown in square brackets

Overall, the majority of the study population followed an omnivorous diet. The proportion of vegetarians and vegans increased among participants aged over 10 years. Detailed numbers for the main study groups and the subgroups selected for matching are presented in the flowchart below (Fig. 1).

The prevalence of vegetarian diets increased with age and was consistently higher among females. In the 0–6-year age group, 1.4% (18/1,257) of children followed a vegetarian diet at least once, rising to 2.7% (28/1,019) among 6–10-year-olds.

Between ages 10 and 13, 6.9% of girls and 3.3% of boys reported vegetarian diets at least once, contributing 59 samples (only visits with reported vegetarian diet). Among 13–15-year-olds, the prevalence rose to 11.6% in girls and decreased to 2.2% in boys (49 and 9 samples, respectively). In the 15–17-year group, 8.4% (46/545) of the subjects followed a vegetarian diet, with females (15.4%) being more than five times as likely as males (2.7%) to do so. This sex difference decreased slightly in the 17–19-year-olds (females: 15.2%, males: 4.2%), with a total of 38 samples from vegetarian participants.

Overall, it clearly shows that girls were more likely to report a vegetarian diet than boys. The number of blood samples of subjects following a vegetarian diet was in total 277 of 7344 samples (3.8%) (157/2,508 participants). According to the restricted age for further statistics (> 10 years), we have 215 blood samples from 128 vegetarians left for analyses.

Vegan diets were rare across all age groups. Up to age 15, prevalence remained below 0.4%. Among 15–17-year-olds, 0.8% of females (2/247) and 0.7% of males (2/298) reported a vegan diet (5 samples). In the 17–19-year group, 2.3% of participants were following a vegan diet (females: 3.2%, males: 1.6%). A total of 21 blood samples were available from individuals following a vegan diet, of which 17 samples from 12 participants aged ≥ 10 years were included in further analyses.

The frequencies of all age groups are listed in detail in Supplementary Table 1.

### Diet and anthropometric data

#### Height-SDS

**Fig. 2:**
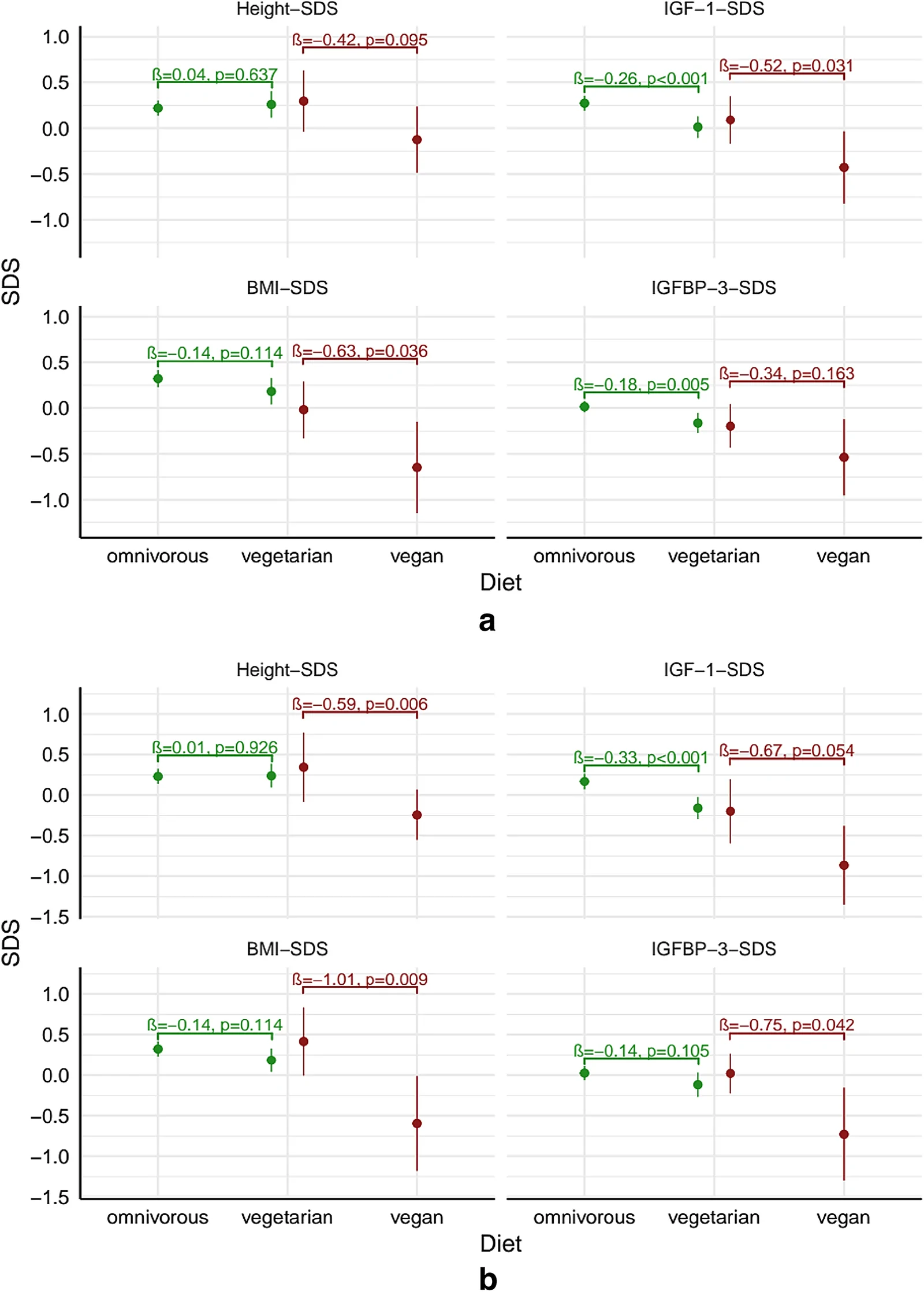
(a) Age-adjusted and (b) TS-adjusted SD-scores for height, BMI, IGF-1 and IGFBP-3 presented asmeans and 95% confidence intervals. Results are based on two pairwise 3:1 matched-controlcomparisons (omnivorous (645 samples) vs. vegetarian (215 samples) and vegetarian (51 samples) vs.vegan (17 samples). Vegetarian groups therefore appear in both comparisons and are shown as twoseparate data points. ß-coefficients and p-values refer to group comparisons.

Vegetarian children showed the highest mean height-SDS, closely followed by omnivorous children. This pattern was consistent regardless of whether models were adjusted for age (Fig. 2a) or pubertal status (Fig. 2b). However, the differences were small and did not reach statistical significance. In contrast, vegan children with − 0.24 height-SDS were significantly shorter than vegetarian children with 0.35 height-SDS when adjusting for pubertal status (β = −0.59, *p* = 0.006). Age-adjusted SD-scores did not differ statistically significant between the groups.

#### BMI-SDS

We observed the highest BMI-SDS in children following an omnivorous diet (0.32 SDS) (Fig. 2a, b). The vegetarian group had a 0.14 lower BMI-SDS (*p* = 0.114). The effect size and p-value are similar in age-adjusted and TS-adjusted models.

Considering both age- and puberty-adjusted SDS, vegan children showed with − 0.63 significantly lower BMI-SDS values than vegetarian children. This effect was more pronounced in the TS–adjusted model, yielding an estimate of β = − 1.01 (*p* = 0.009).

### Diet and growth factors

#### IGF-1

Vegetarians exhibited significantly lower IGF-1 serum concentrations than omnivores. The effect was stronger for TS-adjusted outcomes (ß=−0.33, *p* < 0.001) (Fig. 2b). In contrast, the difference between vegetarians and vegans was significant only for age-adjusted outcome (Fig. 2a), with children on a vegan diet showing 0.52 SDS lower IGF-1 than vegetarians (*p* = 0.031).

#### IGFBP-3

For both age- and pubertal status–adjusted outcomes, vegetarians had lower IGFBP-3 concentrations than omnivores, and vegans exhibited lower IGFBP-3 concentrations than vegetarians. The difference between omnivores and vegetarians reached statistical significance for age-adjusted outcomes (ß=−0.18, *p* = 0.005; 0.02 vs. -0.16 SDS) **(**Fig. 2a**)**. The difference between vegetarians and vegans was only statistically significant in TS–adjusted models (ß=−0.75, *p* = 0.042; 0.02 vs. -0.73 SDS) **(**Fig. 2b**)**.

The results are listed in detail in Supplementary Table 2. Further, we have added the respective comparisons between the coefficients (ß) of both models in Supplementary Table 3. The results of the sensitivity analyses were consistent between models with with exchange and independent correlation structure, which supports the robustness of our findings.

## Discussion

### Prevalence

According to a German survey conducted in 2021, 10% of the general population follow a vegetarian diet, and an additional 2% adhere to a vegan diet [29]. These findings are comparable to the proportions observed in our oldest age group: 9.2% of 17- to 19-year-olds reported following a vegetarian diet at least once, while 2.3% followed a vegan diet.

Several studies have consistently reported a higher prevalence of vegetarianism among females compared to males. Similar to our findings, the Ipsos study observed that more females (6%) followed a vegetarian diet, compared to males (4%) [30]. Regarding adolescents, another study reported a comparable overall prevalence of vegetarianism (10%), with a marked sex difference: 14% of girls versus 7% of boys identified themselves as vegetarians [31]. These findings are also supported by additional research indicating that females are more likely to adopt a vegetarian diet across various demographic groups [32, 33]. In general, women have been shown to adhere more frequently to diets perceived as healthier or more ethically than men [34].

### BMI and height

The literature reports inconsistent associations between dietary patterns and various anthropometric measures. A study by Elliot et al. [35] involving 8907 children, including 248 vegetarians, found no significant association between dietary pattern and BMI or height-for-age score. However, the z-score for height (standardized for age and sex) was lower in vegetarian children, with an height difference of approximately 0.3 cm in three-year-olds. The same study reported an odds ratio of 1.87 for underweight status among vegetarian children, suggesting a higher prevalence of underweight in this group. In our study, vegetarian children also had a lower BMI, while in contrast to Elliot et al., they tended to be slightly taller, although this difference was not statistically significant.

Ambroszkiewicz et al. [36] found no significant differences in BMI, weight, or height between omnivorous and vegetarian normal-weight children. Similarly, Rudloff et al. [37] observed no differences in height or BMI between dietary groups. However, differences in body weight and body fat percentage were noted, with vegetarian children and adolescents having lower values in both parameters.

According to Desmond et al. [38], vegan and vegetarian children aged five to ten years were shorter, but significance was only reached for vegan children. Furthermore, vegans had a lower BMI and a similar lean mass but lower fat indices in all body regions. Likewise, in the VeChi Diet Study [7], one- to three-year-old children who were fed a vegetarian or vegan diet were found to be shorter, lighter, and less physically active than their omnivorous peers. When comparing dietary groups across different age groups, no significant differences were observed in children or young adults. Only during adolescence were vegetarians shorter and lighter than the reference group and had a lower BMI.

Sabaté et al. [39] compared the anthropometric measurements of a large cohort of children in the United States. Vegetarian boys were reported to be, on average, 1.6 cm taller and 1.27 kg lighter. Vegetarian girls were 1.16 kg lighter and had a BMI that was 0.43 points lower. However, it is important to note that the study distinguished between children adhering to the Seventh-Day Adventist (SDA) lifestyle and attending corresponding schools, and those attending public schools. The SDA community largely follows a lacto-ovo-vegetarian diet and abstains from alcohol and nicotine. Additionally, SDA families tend to have a higher socioeconomic status and income compared to families whose children attend public schools. Therefore, the observed anthropometric differences may be partially attributable to these confounding factors.

### IGF-1 and IGFBP-3

There is a notable lack of studies specifically examining the correlation between vegetarian or vegan diets in children and their IGF-1 levels. Most existing research focuses either on adults or on the association of IGF-1 with (animal) protein intake or other nutrients. Nevertheless, several findings from the literature are in line with our results, either directly or indirectly:

Several studies report a positive association between higher protein intake and IGF-1 levels. In one study involving six-month-old infants, those fed a formula with approximately 60% higher protein content were observed to have significantly higher total and free IGF-1 concentrations (*p* < 0.001). Specifically, the increase in total IGF-1 was 24.36 ng/mL in female infants (95% CI 11.40–37.33; *p* < 0.001) compared to the low-protein formula group, whereas the mean difference in male infants was smaller but not statistically significant (11.22 ng/mL; 95% CI 1.85–24.28; *p* = 0.140) [40]. A similar study by Socha et al. reported a 40% increase in IGF-1 levels in children receiving a high-protein formula primarily based on milk protein [41]. Another study showed that an increase of 10 g of daily protein intake in early childhood was associated with a higher BMI z-score, greater DXA-measured lean mass, and higher circulating IGF-1 levels during early adolescence in boys [19]. These correlations were observed only in boys and with protein of animal origin; no associations were reported for girls or plant-based proteins. In vegan women IGF-1 levels were 13% lower than in those following other dietary patterns [21], which has also been linked to a reduced intake of essential amino acids.

These findings are consistent with our statistical results and support an association between vegetarian and vegan dietary patterns and lower IGF-1 concentrations in children, which may be related to differences in dietary composition, including a lower intake of animal proteins.

For IGFBP-3, the available evidence remains limited and inconsistent. In particular, multiple studies did not observe significant associations between dietary patterns and circulating IGFBP-3 concentrations [18, 21, 41, 42] suggesting that IGFBP-3 may be less sensitive to differences in dietary composition than IGF-1.

In contrast, other studies support our results: In female adolescents, a lower intake of animal protein has been associated with reduced circulating concentrations of IGFBP-3 [43]. Similarly, studies in males have shown a positive association between IGFBP-3 levels and the intake of dietary protein, particularly of animal origin [44]. Hoppe et al. observed an increase in IGFBP-3 concentrations with higher milk consumption, whereas no such association was found for meat intake [45]. Consistent with these findings, another study reported elevated IGFBP-3 concentrations after consumption of casein and skimmed milk, in comparison to water [46]. These results align with those of Crowe et al. [18]and Kerver et al. [47], who found positive correlations between dietary calcium intake and IGFBP-3 levels.

## Strengths and limitations

This study provides novel insights into the relationship between diets and growth including corresponding serum concentrations of IGF-1 and IGFBP-3 in children and adolescents. To our knowledge, it is among the first to compare these biomarkers across omnivorous, vegetarian, and vegan diets in a large, population-based pediatric sample. The age range (10–19 years) allowed for detailed age- and sex-stratified analyses. Importantly, the availability of pubertal stage (Tanner stage) enabled adjustments beyond chronological age, offering a more accurate assessment of biomarker levels in the context of biological maturation. Furthermore, standardized blood sample collection and centralized laboratory analysis strengthen the reliability and validity of the biochemical data. Another strength of this study is the high degree of population homogeneity, with 98% of participants being of Caucasian ethnicity. This reduces potential confounding related to ethnic heterogeneity and increases the internal consistency of the findings.

At the same time, this also represents a limitation, as it restricts the generalizability of the results to more ethnically diverse populations. Therefore, the findings should primarily be interpreted within the context of a predominantly White European pediatric cohort. Furthermore, although significant differences were observed after matching, the limited size of the vegan group (*n* = 17) reduces the robustness and generalizability of our conclusions, especially for vegans. Accordingly, borderline-significant findings observed in this group, such as for IGF-1-SDS (β = −0.52, *p* = 0.031), should be interpreted with caution. In addition, the wide confidence intervals observed in comparisons involving vegan participants reflect substantial statistical uncertainty. Therefore, these results should not be overinterpreted. Replication in larger, well-powered cohorts or meta-analyses is required before firm conclusions regarding the specific associations between vegan diets and growth- or endocrine-related outcomes in children can be drawn. In addition, dietary information was based on self-report and collected at each visit, without detailed assessment of diet duration, adherence, or nutrient intake, potentially introducing misclassification bias. Further, the partially small group sizes did not allow to include other factors, such as physical activity, socioeconomic status, or other lifestyle variables in our analyses. Finally, as our study is cross-sectional, causal inferences cannot be drawn, and the possibility of reverse causality — although unlikely in this pediatric context — cannot be entirely excluded. Prospective studies are warranted to further elucidate the directionality of the observed associations.

## Conclusion

Children following a vegetarian diet had a reduced BMI compared with omnivores. Vegans have significantly lower values in height and BMI than vegetarians. We found significantly lower IGF-1 and IGFBP-3 levels in children following both a vegetarian or vegan diet. These findings emphasize the importance of monitoring the nutritional status of children following restrictive diets and critically evaluating dietary guidelines, particularly during phases of rapid growth and development. However, no specific dietary recommendations can be derived from these observational findings. Further studies with more detailed assessment of dietary intake and nutrient composition are needed to better elucidate the underlying mechanisms and to inform evidence-based nutritional recommendations.

## Supplementary Information

Below is the link to the electronic supplementary material.

Supplementary Material 1.

## Data Availability

Data described in the manuscript, code book, and analytic code will be made available upon request pending application and approval. The dataset presented in this article will not be shared publicly because of legal and ethical restrictions. LIFE Child collects potentially sensitive information. The informed consent provided by study participants does not cover the publication of this data. Furthermore, the LIFE data protection policy requires all researchers, both internal and external, who wish to access the data to sign a project agreement. Researchers interested in accessing data from the LIFE Child study can reach out by emailing forschungsdaten@medizin.uni-leipzig.de.

## Abbreviations

BMI: Body mass index
IGF-1: Insulin-like growth factor 1
IGFBP-3: Insulin-like growth factor-binding protein 3
SDS: Standard deviation score
TS: Tanner stages
