# Supplementary material for: Growth parameters and IGF-1 axis biomarkers differ among omnivorous, vegetarian, and vegan children
Source: Eur J Nutr. 2026 Aug 29;65(6):245. doi: 10.1007/s00394-026-04085-6 (PMC13526060; doi:10.1007/s00394-026-04085-6)
Supplement: Supplementary file 1 [file 394_2026_4085_MOESM1_ESM.pdf]

age group	(0,3], N = 509 [450]		(3,6], N = 1152 [807]		(6,10], N = 1936 [1019]		(10,13], N = 1534 [927]		(13,15], N = 1008 [694]		(15,17], N = 768 [545]		(17,19], N = 437 [348]	
**sex**	**female N = 253 [228]**	**male N = 256 [222]**	**female N = 541 [372]**	**male N = 611 [435]**	**female N = 865 [467]**	**male N = 1,071 [552]**	**female N = 698 [418]**	**male N = 836 [509]**	**female N = 485 [337]**	**male N = 523 [357]**	**female N = 345 [247]**	**male N = 423 [298]**	**female N = 199 [157]**	**male N = 238 [191]**
**age (years)**														
Median [Q1-Q3]	2.0 [2.0-2.8]	2.0 [2.0-2.9]	4.8 [4.0-5.1]	4.6 [4.0-5.2]	8.0 [7.0-9.0]	8.1 [7.1-9.0]	11.5 [10.8-12.2]	11.4 [10.8-12.2]	14.0 [13.6-14.5]	14.0 [13.5-14.5]	15.9 [15.6-16.5]	15.9 [15.5-16.4]	17.7 [17.4-18.0]	17.7 [17.3-18.0]
Mean±SD	2.28±0.43	2.32±0.47	4.55±0.89	4.52±0.90	8.03±1.17	8.07±1.17	11.54±0.86	11.51±0.85	14.01±0.57	13.99±0.59	15.99±0.58	15.94±0.56	17.72±0.42	17.69±0.42
**BMI-SDS**														
Median [Q1-Q3]	0.2 [-0.4- 0.8]	0.3 [-0.4- 0.8]	-0.1 [-0.6-0.6]	0.0 [-0.6-0.6]	-0.3 [-0.8-0.4]	-0.2 [-0.8-0.4]	-0.1 [-0.7,-0.6]	-0.2 [-0.8-0.6]	0.1 [-0.5-0.8]	-0.0 [-0.7-0.8]	0.3 [-0.4-1.0]	0.1 [-0.6-0.8]	0.3 [-0.3-1.0]	0.2 [-0.7-0.9]
Mean±SD	0.19±0.87	0.22±0.92	-0.03±0.8	-0.01±0.83	-0.15±0.98	-0.16±0.96	0.05±1.13	-0.03±1.09	0.24±1.12	0.07±1.14	0.40±1.19	0.18±1.13	0.51±1.39	0.12±1.15
**vegan**	1 (0.4%) [1]	0 (0%) [0]	1 (0.2%) [1]	2 (0.3%) [2]	0 (0%) [0]	0 (0%) [0]	1 (0.1%) [1]	0 (0%) [0]	0 (0%) [0]	1 (0.2%) [1]	3 (0.9%) [2]	2 (0.5%) [2]	7 (3.5%) [5]	3 (1.3%) [3]
**vegetarian**	5 (2.0%) [3]	4 (1.6%) [4]	8 (1.5%) [5]	8 (1.3%) [6]	25 (2.9%) [19]	11 (1.0%) [9]	37 (5.3%) [30]	22 (2.6%) [17]	49 (10%) [39]	9 (1.7%) [8]	52 (15%) [38]	9 (2.1%) [8]	29 (15%) [24]	9 (3.8%) [8]
**TS**														
1	253 (100%) [228]	256 (100%) [222]	534 (100%) [370]	599 (100%) [432]	748 (94%) [436]	1,040 (98%) [546]	119 (20%) [103]	302 (51%) [254]	0 (0%) [0]	4 (1.4%) [4]				
2					47 (5.9%) [46]	18 (1.7%) [18]	216 (36%) [193]	204 (35%) [180]	10 (2.2%) [10]	39 (14%) [37]				
3					1 (0.1%) [1]	1 (<0.1%) [1]	156 (26%) [139]	63 (11%) [59]	51 (11%) [47]	81 (29%) [75]	1 (0.3%) [1]	10 (4.6%) [10]		
4							19 (3.2%) [19]	18 (3.0%) [18]	20 (4.4%) [19]	102 (36%) [91]	1 (0.3%) [1]	53 (24%) [52]	0 (0%) [0]	3 (2.5%) [3]
5							82 (14%) [74]	4 (0.7%) [4]	369 (82%) [269]	54 (19%) [53]	335 (99%) [242]	156 (71%) [126]	199 (100%) [157]	117 (98%) [107]
NA			7	12	69[64]	12[12]	106[93]	245[216]	35[34]	243[209]	8[8]	204[171]	0	118[110]

Supplementary Table 1 Frequencies of participants by age group and sex. Age and BMI-SDS are presented as median (Q1-Q3) and means±SD; Tanner stages and categorical variables as absolute and relative frequencies (n (%)). Values outside brackets indicate the number of visits, values in square brackets refer to the number of unique participants.

		Omnivorous vs. Vegetarian			Vegetarian vs. Vegan		
**Measurements**	adjustment	omnivorous	vegetarian	P-value	vegetarian	vegan	P-value
**BMI-SDS**	Age	0.32 (0.23; 0.41)	0.18 (0.04; 0.33)	0.114	-0.19 (-0.33; 0.29)	-0.65 (-1.14; -0.15)	0.036
	TS	0.32 (0.23; 0.41)	0.18 (0.04; 0.33)	0.114	0.41 (0.0; 0.84)	-0.60 (-1.18; -0.01)	0.009
**Height-SDS**	Age	0.22 (0.14; 0.30)	0.26 (0.12; 0.40)	0.637	0.30 (-0.04; 0.63)	-0.13 (-0.49; 0.24)	0.095
	TS	0.23 (0.14; 0.33)	0.24 (0.09;0.39)	0.926	0.35 (-0.08; 0.77)	-0.24 (-0.55; 0.07)	0.006
**IGF-1-SDS**	Age	0.27 (0.19; 0.35)	0.01 (-0.11; 0.13)	<0.001	0.09 (-0.17; 0.35)	-0.43 (-0.83; -0.03)	0.031
	TS	0.17 (0.07; 0.27)	-0.16 (-0.29; -0.03)	<0.001	-0.20 (-0.59; 0.20)	-0.87 (-1.36; -0.38)	0.054
**IGFBP-3-SDS**	Age	0.01 (-0.05; 0.07)	-0.16 (-0.27; -0.05)	0.005	-0.20 (-0.43; 0.04)	-0.54 (-0.95; -0.12)	0.163
	TS	0.02 (-0.06; 0.11)	-0.12 (-0.27; 0.03)	0.105	0.02 (-0.23; 0.26)	-0.73 (-1.30; -0.15)	0.042

Supplementary Table 2 SD- values with 95% confidence intervals (conf min; conf max) corresponding to Figures 1a and 1b

	**Age-adjusted**				**TS-adjusted**			
	**omnivorous vs. vegetarian**		**vegetarian vs. vegan**		**omnivorous vs. vegetarian**		**vegetarian vs. vegan**	
**Outcome**	diff_exch	diff_indep	diff_exch	diff_indep	diff_exch	diff_indep	diff_exch	diff_indep
**IGF-1**	-0.261	-0.261	-0.520	-0.506	-0.327	-0.334	-0.671	-0.574
**IGFBP-3**	-0.176	-0.176	-0.341	-0.342	-0.141	-0.163	-0.748	-0.605
**Height-SDS**	0.039	0.040	-0.421	-0.440	0.008	0.028	-0.588	-0.656
**BMI-SDS**	-0.138	-0.140	-0.628	-0.629	-0.138	-0.157	-1.010	-1.001

Supplementary Table 3 Comparison of β-coefficient estimates (diff_exch) from the main analysis and the sensitivity analysis (diff_indep) using an independence correlation structure (cluster-robust OLS),
